# β_2_-Adrenergic Receptors Chaperone Trapped Bitter Taste Receptor 14 to the Cell Surface as a Heterodimer and Exert Unidirectional Desensitization of Taste Receptor Function*

**DOI:** 10.1074/jbc.M116.722736

**Published:** 2016-06-24

**Authors:** Donghwa Kim, Susan H. Pauer, Hwan M. Yong, Steven S. An, Stephen B. Liggett

**Affiliations:** From the Departments of ‡ Medicine and; ‖ Molecular Pharmacology and Physiology, and; the § Center for Personalized Medicine and Genomics, University of South Florida Morsani College of Medicine, Tampa, Florida 33612 and; the ¶ Department of Environmental Health Sciences, The Johns Hopkins University, Bloomberg School of Public Health, Baltimore, Maryland 21205

**Keywords:** asthma, chronic obstructive pulmonary disease (COPD), cyclic AMP (cAMP), dimerization, G protein, receptor internalization, smooth muscle, bitter taste receptor

## Abstract

Bitter taste receptors (TAS2Rs) are G-protein-coupled receptors now recognized to be expressed on extraoral cells, including airway smooth muscle (ASM) where they evoke relaxation. TAS2Rs are difficult to express in heterologous systems, with most receptors being trapped intracellularly. We find, however, that co-expression of β_2_-adrenergic receptors (β_2_AR) in HEK-293T routes TAS2R14 to the cell surface by forming receptor heterodimers. Cell surface TAS2R14 expression was increased by ∼5-fold when β_2_AR was co-expressed. Heterodimer formation was shown by co-immunoprecipitation with tagged receptors, biomolecular fluorescence complementation, and merged confocal images. The dynamic nature of this interaction was shown by: a gene-dose relationship between transfected β_2_AR and TAS2R14 expression, enhanced (up to 3-fold) TAS2R14 agonist stimulation of [Ca^2+^]*_i_* with β_2_AR co-transfection, ∼53% decrease in [Ca^2+^]*_i_* signaling with shRNA knockdown of β_2_AR in H292 cells, and ∼60% loss of [Ca^2+^]*_i_* responsiveness in βAR knock-out mouse ASM. Once expressed on the surface, we detected unidirectional, conformation-dependent, interaction within the heterodimer, with β_2_AR activation rapidly uncoupling TAS2R14 function (∼65% desensitization). Cross-talk was independent of β_2_AR internalization and cAMP/PKA, and not accompanied by TAS2R14 internalization. With prolonged β-agonist exposure, TAS2R14 internalized, consistent with slow recycling of naked TAS2R14 in the absence of the heterodimeric milieu. In studies of ASM mechanics, rapid cross-talk was confirmed at the physiologic level, where relaxation from TAS2R14 agonist was decreased by ∼50% with β-agonist co-treatment. Thus the β_2_AR acts as a double-edged sword: increasing TAS2R14 cell surface expression, but when activated by β-agonist, partially offsetting the expression phenotype by direct receptor:receptor desensitization of TAS2R14 function.

## Introduction

Bitter taste receptors (TAS2R)^2^ were initially discovered on taste buds and were thought to have evolved as a mechanism for avoidance of toxic plants (1, 2). However, we found that certain TAS2Rs (subtypes 10, 14, and 31) are expressed on human airway smooth muscle (HASM), and when activated result in marked HASM relaxation and bronchodilation (3, 4). This has brought forth the concept of HASM TAS2Rs being targets for novel agonists in the treatment of asthma and chronic obstructive pulmonary disease (4, 5). TAS2Rs are also expressed on a number of other extraoral cell types, suggesting a previously unrecognized chemo-sensory system that might be exploited for drug development (5, 6). TAS2Rs in taste cells signal by binding to the G-protein gustducin, whose βγ subunit activates phospholipase C, generating inositol 1,4,5-trisphosphate, which activates an endoplasmic reticulum inositol 1,4,5-trisphosphate receptor resulting in an increase in intracellular Ca^2+^ ([Ca^2+^]*_i_*) (1). There is a divergence in signaling between taste cells and HASM at this juncture. In taste cells, the TAS2R-derived [Ca^2+^]*_i_* activates a transient receptor potential channel, causing membrane depolarization, release of neurotransmitter, and subsequent activation of the Type III cell, which through sensory nerves communicates to the central nervous system. In HASM, the expressed TAS2Rs act directly to relax the muscle through a non-cAMP dependent mechanism, involving [Ca^2+^]*_i_* modulation (3). Indeed the efficacy of some TAS2R agonists is greater than full β_2_-adrenergic receptor (β_2_AR) agonists (4), which are the mainstay of treating bronchospasm in asthma and chronic obstructive pulmonary disease. The relaxation from activation of β_2_AR expressed on HASM is due to coupling of these receptors to G_s_, with generation of cAMP, and a protein kinase A-dependent mechanism of relaxation (7). Given the extensive relaxation evoked from TAS2Rs, and the different mechanisms by which TAS2Rs and β_2_ARs relax HASM, the idea of using agonists for these receptors singly or in combination has been put forward as a way to optimize therapy (5).

The 25 TAS2Rs have been historically difficult to heterologously express on the cell membrane of model cells (8), which has been an impediment for further investigation of their signaling properties. However, in the process of expressing the TAS2R14 subtype with the β_2_AR, we found an increase in expression in HEK-293T cells. This led to the hypothesis that TAS2R14 and β_2_AR form a heterodimer in the cytosol, and TAS2R14 cell surface expression is facilitated by the β_2_AR component. In this report, we show that transfected TAS2R14 is predominately trapped in the cytosol in the absence of co-transfected β_2_AR, and that β_2_AR acts as a chaperone to facilitate TAS2R14 membrane insertion and functional coupling. This translocation is due to the formation of TAS2R14:β_2_AR heterodimers. We show that the heterodimeric unit is stable at the cell surface, and identify a mechanism of unidirectional cross-talk between the two receptors that uncouples TAS2R signaling. Physiologic consequences of the heterodimer and the cross-talk are confirmed in studies of ASM cell mechanics. Taken together, we provide new insight into how TAS2R14 is expressed and regulated by β_2_AR, and potential interactions between the receptors that may impinge on therapeutic efficacy.

## Results

### 

#### 

##### Co-expression of β_2_AR Enhances Cell Membrane TAS2R14 Expression

**FIGURE 1.**
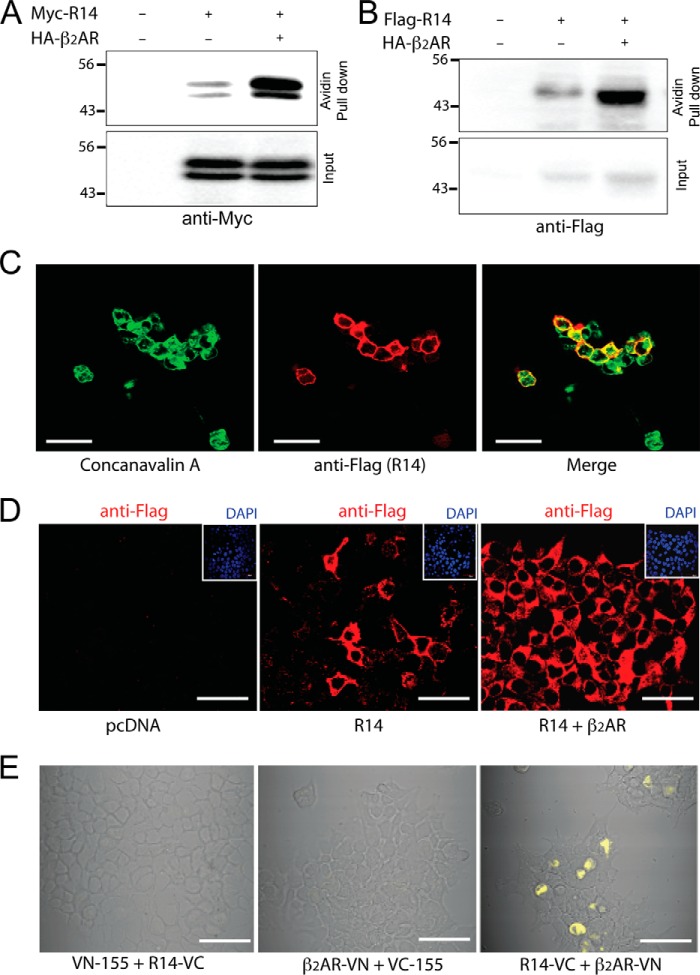
**Enhanced expression of TAS2R14 by β_2_AR co-transfection**. HEK-293T cells were transfected with the epitope-tagged TAS2R14 construct (see “Experimental Procedures”) in the absence or presence of co-transfection with β_2_AR (*A–E*). Cells were exposed to biotin and cell surface proteins were collected using avidin-immobilized agarose beads. The released proteins were subjected to SDS-PAGE and immunoblotted with Myc (*A*) or FLAG antibody (*B*). In *C,* the cell membrane is identified by concanavalin A (*green* signal) and TAS2R by FLAG antibody (*red* signal). Merged images reveal TAS2R14 localizes to the cell surface (*yellow* signal). In *D*, increased TAS2R14 expression was also observed by confocal imaging (×400), when β_2_AR was co-transfected. Confocal images (×400) from BiFC studies (*E*), with the indicated regions of Venus, shows complementation only when they are fused to the two receptors, indicative of TAS2R14-β_2_AR interaction. Results are representative of 3–5 experiments. *Bars*: *C* = 30 μm; *D* and *E* = 40 μm.

To begin to address potential TAS2R:β_2_AR interactions, we attempted to heterologously express the receptors in HEK-293T cells. Our initial approach to transfect these cells with FLAG-TAS2R14 in pcDNA resulted in very little expression in the cytosol or on the cell membrane, as has been documented by others (2, 8). Extension of the short amino terminus with the rat somatostatin receptor 3 amino terminus, and the C terminus with a herpes simplex virus glycoprotein D epitope (a common approach in the TAS2R field, which has been reported to provide for some degree of expression) (2) did not result in consistently detectable expression in our hands. When we added a cleavable leucine-rich N-terminal peptide, termed Lucy (9), to the aforementioned construct (Lucy-Flag-rsstr3-TAS2R14-HSV), expression over background was achieved as determined by Western blotting analysis using FLAG or Myc antibodies (Fig. 1, *A* and *B*). However, when the above TAS2R14 construct was co-transfected with β_2_AR in pcDNA, a substantial increase in cell surface expression of TAS2R14 was observed (Fig. 1, *A* and *B*). For these studies, cells were transfected with FLAG- or Myc-tagged TAS2R14, in the absence or presence of co-transfection with β_2_AR, and 48 h later the intact cells were treated with biotin. After purification with avidin immobilized on agarose beads to isolate membrane-bound proteins, the proteins were subjected to SDS-PAGE and immunoblotting with Myc or FLAG antibody. As depicted in Fig. 1, *A* and *B*, TAS2R14 cell surface expression was increased when HEK-293T cells were co-transfected with β_2_AR. In 4 such experiments, the fold-increase of cell surface TAS2R14 when β_2_AR was co-transfected was determined to be 5.7 ± 0.96-fold (*p* < 0.01 *versus* TAS2R14-transfected). Confocal imaging of co-transfected cells using the FLAG antibody to identify TAS2R14 (*red* signal) and concanavalin A to delineate the cell membrane (*green* signal) confirmed membrane association of the expressed TAS2R14 (*yellow* signal) (Fig. 1*C*). Additional confocal imaging studies (Fig. 1*D*) were concordant with the biotinylation assays. In the absence of β_2_AR co-transfection, TAS2R14 expression (*red* signal) was found in ∼20% of cells, but rarely at the cell surface. However, when co-transfected with β_2_AR, most cells were found to express TAS2R14 and its cell surface expression was readily apparent, amounting to 80% of the total (intracellular + cell surface) TAS2R14 expression (Fig. 1*D*).

##### β_2_AR and TAS2R14 Form Heterodimers Facilitating TAS2R14 Expression

**FIGURE 2.**
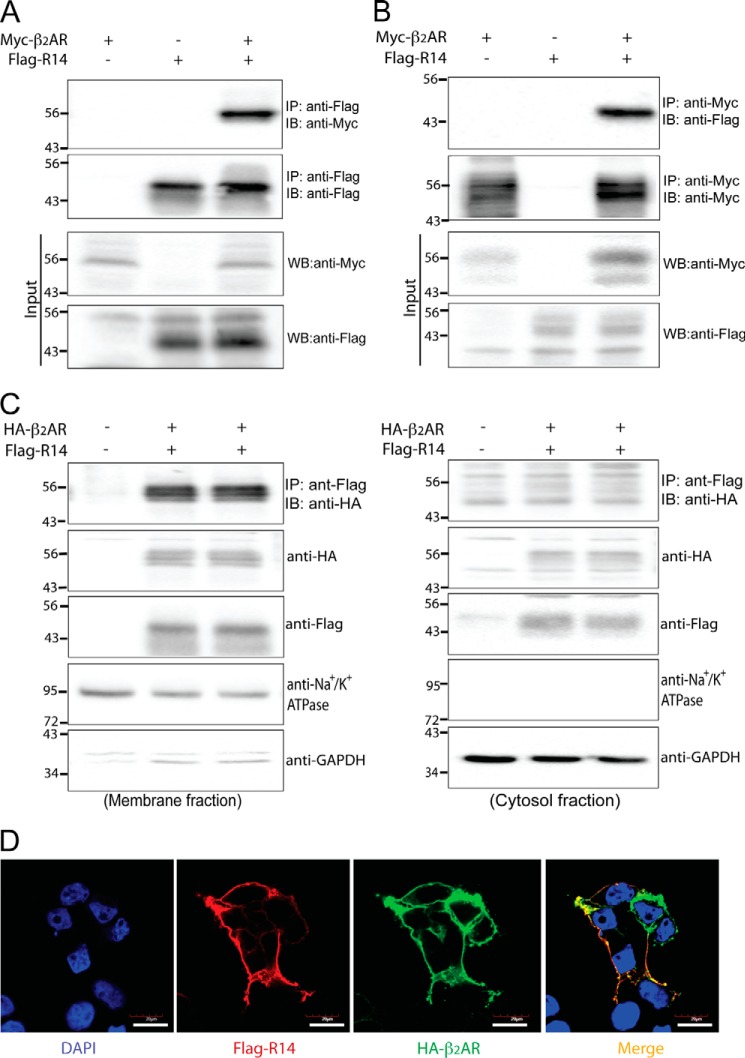
**TAS2R14 and β_2_AR co-immunoprecipitate and co-localize.** HEK-293T cells were transfected with Myc-β_2_AR and FLAG-TAS2R14 (*A*). IP with FLAG antibodies followed by SDS-PAGE and immunoblotting (*IB*) with Myc antibodies only showed a band at the expected *M*_r_ when both receptors were transfected. Immunoblotting of the FLAG immunoprecipitates showed signals when FLAG-TAS2R14 was transfected. The inputs to the IP shown in the lower two sections confirm expression of the individual receptors in the cell lysates (10 μg) as indicated. In *B*, a similar experiment was performed except the IP was with Myc antibody and the IB was with FLAG antibody. For *C*, co-IP studies with HA- or FLAG-tagged receptors were performed with protein from either membrane fractions (200 μg) or cytosolic fractions (250 μg), and revealed the TAS2R14:β_2_AR heterodimer in both fractions. Confocal imaging (*D*, ×600) of HA-β_2_AR and FLAG-TAS2R14 co-transfected cells shows cell surface expression of each receptor (*green* and *red*, respectively) and co-localization (*yellow*) after merging. Results are representative of 4–6 independent experiments. *Bar* = 20 μm.

These results suggested that the β_2_AR protein promotes TAS2R14 expression and/or cell surface integration. Biomolecular fluorescence complementation (BiFC) studies were carried out on live HEK-293T cells (Fig. 1*E*) transfected with the amino terminus of Venus (VN) fused to the carboxyl terminus of β_2_AR (termed VN-β_2_AR) and the carboxyl terminus of Venus (VC) fused to carboxyl terminus of TAS2R14 (termed R14-VC). When VN without β_2_AR was co-transfected with R14-VC there was no fluorescence. Similar findings were observed when β_2_AR-VN was co-transfected with VC without R14. These results are consistent with the notion that overexpression of the Venus proteins in the absence of fusion to both receptor components is insufficient to result in complementation. Fig. 1*E* shows a fluorescent signal that includes intracellular and cell surface components only when β_2_AR-VN and TAS2R14-VC were co-transfected, indicating a close association between the two receptors with reconstitution of the fluorescent chromophore of Venus. Additional studies were performed using co-immunoprecipitation of extracts from cells transfected with FLAG-TAS2R14, Myc-β_2_AR, or both. As shown in Fig. 2*A*, immunoprecipitation with anti-FLAG followed by immunoblotting with anti-Myc resulted in a TAS2R14 signal of the appropriate *M*_r_ only when both receptors were transfected together. Similar results were found when extracts were immunoprecipitated with anti-Myc and immunoblotted with anti-FLAG (Fig. 2*B*). In additional studies, membrane and cytosolic protein fractions were derived from the aforementioned cells, and subjected to co-immunoprecipitation and immunoblotting. TAS2R14-β_2_AR interactions were found in both compartments (Fig. 2*C*), consistent with the BiFC results of Fig. 1*E*. The BiFC and co-immunoprecipitation studies indicate the formation of a TAS2R14:β_2_AR heterodimer, and together with studies from Fig. 1*A,* suggests that the β_2_AR component acts as a chaperone to facilitate TAS2R14 membrane expression. Confocal imaging (Fig. 2*D*) of HEK-293T cells transfected with FLAG-TAS2R14 showed little cell surface expression (*red* signals) as was also shown in Fig. 1*D*. Co-transfection with HA-β_2_AR showed the expected cell surface expression of this receptor (*green* signals). When FLAG-TAS2R14 and HA-β_2_AR were co-transfected, substantial TAS2R14 expression on the cell surface was identified (Fig. 2*D*). Merged signals from both receptors (*yellow*) showed association of TAS2R14 and β_2_AR at the cell surface and within the cell (Fig. 2*D*).

##### Heterologously Expressed TAS2R14 Signal in Response to TAS2R Agonists

**FIGURE 3.**
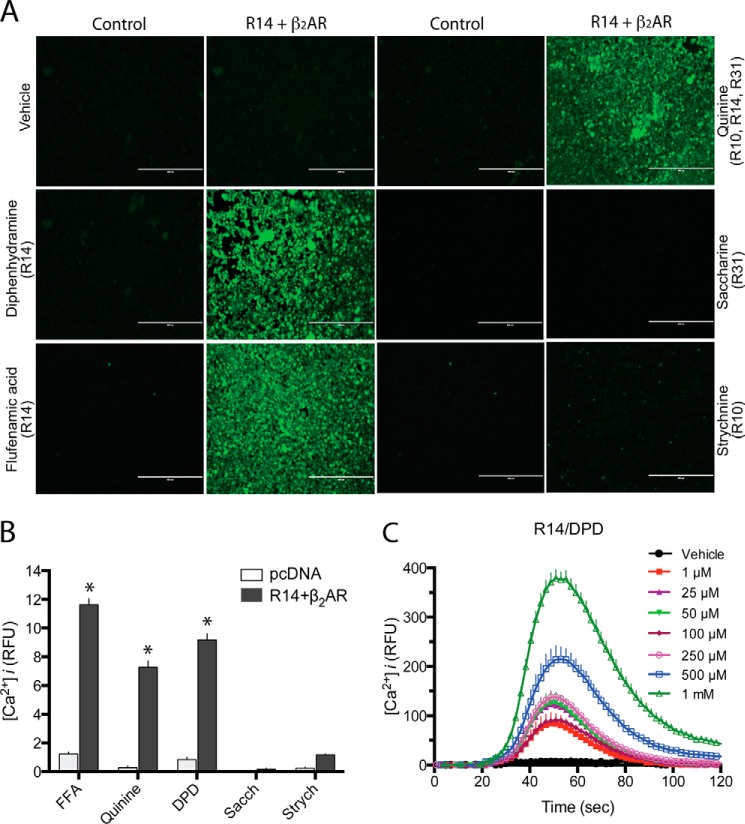
**TAS2R14 expressed on HEK-293T cells couple to [Ca^2+^]*_i_* release.** Cells transfected with Gα16/G44 with TAS2R14 and β_2_AR were loaded with Fluo-4 and [Ca^2+^]*_i_* quantitated by fluorescent microscopy (*A* and *B*) or by a fluorescence based plate based assay (see “Experimental Procedures”) (*C*). Control pcDNA-transfected cells showed a minimal to no response to TAS2R agonists (*A* and *B*). TAS2R14 + β_2_AR cells responded to the TAS2R14 agonists quinine (*QUI*), DPD, and FFA, but not the TAS2R10 agonist strychnine or the TAS2R31 agonist saccharin. *, *p* < 0.01 *versus* pcDNA-transfected, *n* >500 cells imaged per condition. A representative DPD dose-response for stimulation of [Ca^2+^]*_i_* is shown in *C*. Magnification = ×20, *bar* = 400 μm.

**FIGURE 4.**
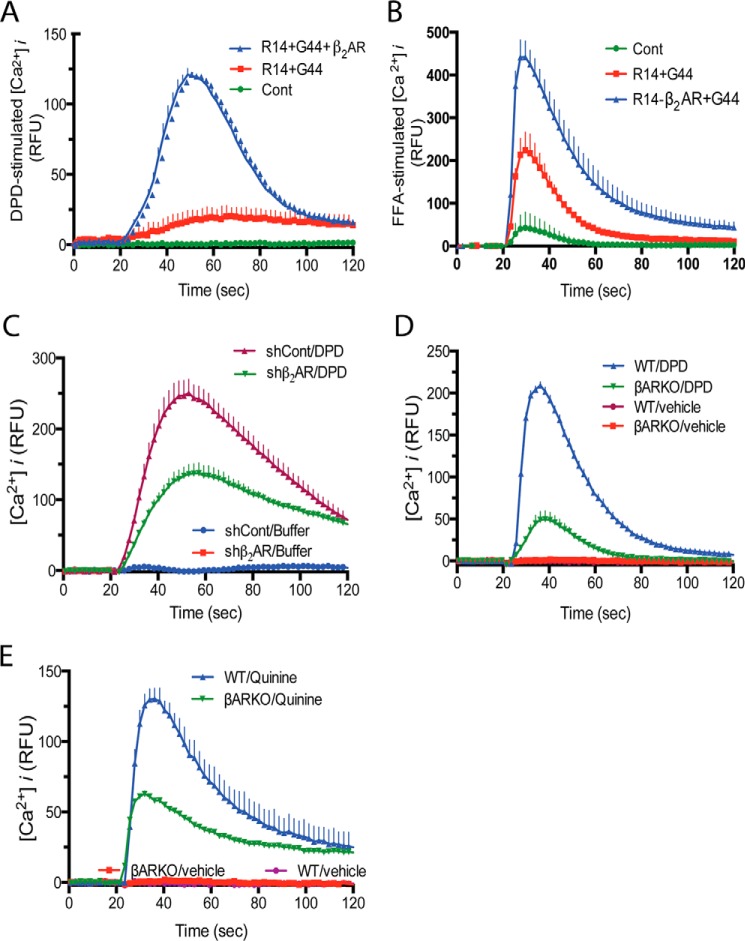
**β_2_AR expression dynamically regulates TAS2R14 functional expression.** HEK-293T cells were transfected with TAS2R14 + G_α_16/G44, without or with β_2_AR (*A* and *B*). The [Ca^2+^]*_i_* response to the TAS2R14 agonists DPD and FFA are increased when β_2_AR is co-transfected, consistent with the increased expression of TAS2R14 (Fig. 1, *A* and *B*). In 4 experiments, the [Ca^2+^]*_i_* stimulation was increased by 2.4 ± 0.11 and 2.1 ± 0.12, respectively, when β_2_AR was co-expressed (*p* < 0.01 *versus* absence of β_2_AR). In *C*, H292 cells, which endogenously express TAS2R14 and β_2_AR were transfected with β_2_AR shRNA (or sh-control) and treated with vehicle or the TAS2R14 agonist DPD. Knockdown of β_2_AR by β_2_AR shRNA resulted in decreased TAS2R14-mediated [Ca^2+^]*_i_* signaling. In *D* and *E*, βAR knock-out mouse (10) ASM cells (which express no detectable βAR) were challenged with TAS2R agonists and revealed >50% reduction in TAS2R-stimulated [Ca^2+^]*_i_*. Results are from 4 representative experiments.

Functional signaling of TAS2R14 was ascertained by fluorescent microscopy and by a plate-based fluorescent assay of Fluo-4-loaded cells. Cells were transfected with the chimeric G-protein Gα16/G44 and pcDNA, and TAS2R14 + β_2_AR. In the imaging studies, β_2_AR + TAS2R14 co-transfected cells showed no response to vehicle, nor did pcDNA-transfected (control) cells show a [Ca^2+^]*_i_* response (Fig. 3*A*) to multiple TAS2R agonists. However, increases in [Ca^2+^]*_i_* were observed for quinine, which activates subtypes 10, 14, and 31, and the TAS2R14 agonists diphenhydramine (DPD) and flufenamic acid (FFA). In contrast, bitter taste receptor agonists for TAS2R31 (saccharin) and TAS2R10 (strychnine) caused no increase in [Ca^2+^]*_i_*. Taken together, these data indicate the expected agonist specificity for TAS2R14 (Fig. 3*B*) (2). In the plate-based studies, co-transfected cells showed the expected dose-responses (2) to DPD (Fig. 3*C*) and quinine (data not shown). Furthermore, cells transfected with TAS2R14, β_2_AR, and Gα16/G44 showed a significant increase in [Ca^2+^]*_i_* response to DPD and FFA (Fig. 4, *A* and *B*) compared with the TAS2R14 + G*_i_*16/G44 cells, consistent with the increase in cell surface TAS2R14 expression evoked by β_2_AR as observe by the biotinylation assay (Fig. 1, *A* and *B*), and the confocal imaging (Figs. 1*D* and 2*D*). In these experiments, expression of β_2_AR compared with control pcDNA did not decrease TAS2R14 mRNA levels as determined by quantitative PCR (4.1 ± 0.12 *versus* 3.5 ± 0.08 units, respectively, *n* = 4, *p* > 0.05).

We further confirmed the dynamic nature of β_2_AR in controlling TAS2R14 expression using two approaches. First, we decreased β_2_AR expression using small hairpin RNA (shRNA) targeting β_2_AR in H292 cells, a mucoepithelial cell line that endogenously expresses β_2_AR and TAS2R14. β_2_AR-shRNA stable transfection caused an 81 ± 2.2% (*n* = 4) decrease in β_2_AR protein expression (data not shown) compared with scrambled control shRNA. In [Ca^2+^]*_i_* assays, this decrease in β_2_AR was associated with a 46 ± 5.6% decrease in DPD-stimulated [Ca^2+^]*_i_* compared with scrambled shRNA (*n* = 4, *p* < 0.01, Fig. 4*C*). These results are consistent with the effects observed when TAS2R14 and β_2_AR are co-transfected in HEK-293T cells, and also indicated that the phenotype is present in cells that endogenously express both receptors. Given our interest in TAS2Rs on ASM as novel targets for treating bronchospasm, we also examined [Ca^2+^]*_i_* responses from cultured ASM cells derived from trachea of WT mice or mice with deleted β_1_AR and β_2_AR genes (βAR-KO) (10), with the expectation that TAS2R expression (and thus the cellular response to agonist) would be reduced in the βAR-KO ASM cells because β_2_AR was not encoded and thus not available to facilitate TAS2R14 expression at the cell surface. Indeed, the βAR-KO mouse ASM cells displayed a 62 ± 9.3% decrease (*n* = 3, *p* < 0.01) in the [Ca^2+^]*_i_* response to DPD compared with the WT ASM cells (Fig. 4*D*). The quinine response was also significantly less in βAR-KO *versus* the WT mouse ASM cells (Fig. 4*E*).

##### Functional Consequences of the TAS2R14:β_2_AR Heterodimeric Complex

**FIGURE 5.**
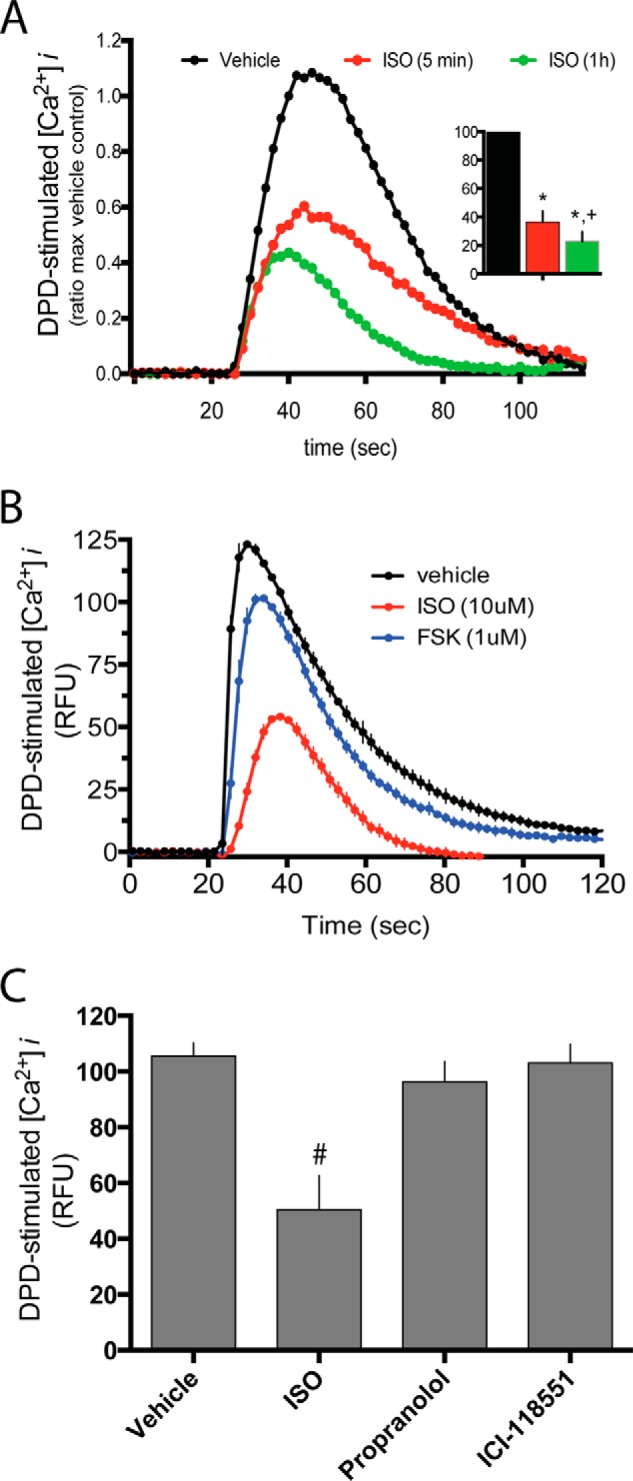
**Activation of β_2_AR within the β_2_AR:TAS2R14 heterodimer uncouples TAS2R14 signaling.** HASM cells were treated with the β-agonist ISO (10 μm) for 5 min or 1 h, and then cells were challenged with the TAS2R14 agonist DPD (250 μm) with immediate quantitation of [Ca^2+^]*_i_* release (*A*). Tracings are from a representative experiment performed in triplicate and the *inset* is mean ± S.E. of 4 experiments. *, *p* < 0.01 *versus* vehicle; +, *p* < 0.05 *versus* 5 min. In *B*, cells were treated with ISO or the cAMP stimulator forskolin (FSK, 10 μm) for 5 min and challenged with DPD as in *A*. Results shown are from a representative experiment of 4 performed, which showed no significant loss (10 ± 8%, *p* > 0.05) of DPD-stimulated [Ca^2+^]*_i_* from forskolin treatment. In *C* cells were treated with the agonist ISO (10 μm), the neutral antagonist propranolol (10 μm), or the β_2_AR inverse agonist ICI118551 (10 μm) for 5 min and then challenged with 250 μm of the TAS2R14 agonist DPD. There was no change in TAS2R14 function with ICI118551 or propranolol pretreatment. #, *p* < 0.01 *versus* vehicle, *n* = 4.

Taken together, these data show, in several cell types using multiple approaches, that the enhancement of TAS2R14 cellular signaling by β_2_AR is due to increased TAS2R14 cell surface expression. We next addressed the potential interactions of the two receptors once the heterodimer is inserted into the membrane at the cell surface. HASM cells were treated with agonist for one receptor for 5 min or 1 h, then the other receptor was activated and function of the second receptor ([Ca^2+^]*_i_* or cAMP) quantitated. The 5-min time point is adequate for agonist engagement of the receptors, which tests whether a conformational change in one receptor influences the conformation/function of the other receptor, a concept that we refer to as “direct receptor:receptor cross-talk.” This time period is also adequate for generation of the immediate second messengers and potential subsequent feedback, which we addressed as a potential mechanism as indicated below. The 1-h pre-exposure is a time period where significant intracellular trafficking and interactions with other proteins may occur, which are distal events after immediate receptor activation, and thus includes other types of regulation as opposed to direct receptor-receptor interaction. To test the effect of activation of β_2_AR on TAS2R14 function, cells were treated with isoproterenol (ISO) for the indicated times followed by DPD or vehicle challenge and immediate measurement of stimulated [Ca^2+^]*_i_* (Fig. 5*A*). Under these conditions, a 64 ± 7.6% decrease in DPD-stimulated [Ca^2+^]*_i_* was observed after ISO exposure of 5 min, and a 78 ± 6.9% decrease (*n* = 4, *p* < 0.05 *versus* 5 min) with the 1 h treatment with ISO (Fig. 5*A*). ISO exposure for 1 h did not decrease TAS2R14 mRNA compared with vehicle control (3.5 ± 0.29 *versus* 3.5 ± 0.52 units, respectively, *p* > 0.05). The loss of TAS2R14 function by β-agonist exposure was not replicated by exposing cells to forskolin, which also increases intracellular cAMP (Fig. 5*B*), indicating that the desensitization of TAS2R14 by β_2_AR is not due to cAMP, or cAMP-promoted PKA activation, with negative feedback to TAS2R14. In contrast, engagement of β_2_AR with the neutral antagonist propranolol, or the β_2_AR-specific inverse agonist ICI118551, had no effect on TAS2R14-stimulated [Ca^2+^]*_i_* (Fig. 5*C*). The relatively rapid loss of TAS2R14 function, the independence from cAMP, and the lack of an effect with neutral or inverse agonists, suggested that there is a direct receptor to receptor interaction imposed on TAS2R when β_2_AR conformation is stabilized by an agonist, leading to a less favorable conformation for TAS2R14 to couple to G-protein.

**FIGURE 6.**
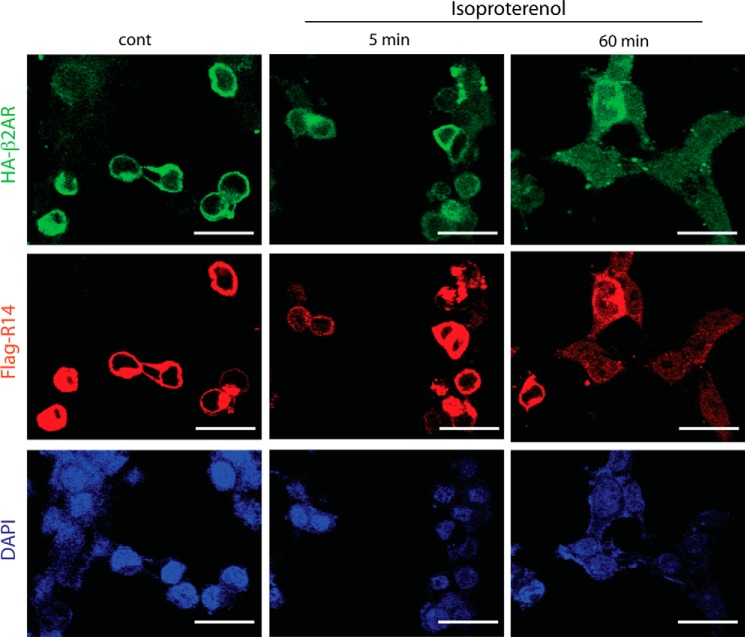
**β_2_AR and TAS2R14 do not co-internalize with ISO exposure.** HEK-293T cells co-transfected with HA-β_2_AR and FLAG-TAS2R14 were imaged by fluorescence microcopy at baseline and after 5 min and 1 h exposure to 10 μm ISO. β_2_AR (*green* signal) at 5 min showed a more diffuse receptor signal at the cell surface, and the presence of intracellular receptor signals compared with control, which is prototypical of β_2_AR internalization. By 1 h, a substantial intracellular β_2_AR signal is observed with a readily apparent decrease in cell surface expression. In contrast, the distribution of TAS2R14 (*red* signal) was unchanged at 5 min, consistent with an absence of internalization. But by 1 h of ISO exposure TAS2R14 consistently showed intracellular receptors. Quantitation is shown in Fig. 7*A*. Images are from a single experiment representative of 5 performed. Magnification = ×600, *bar* = 30 μm.

**FIGURE 7.**
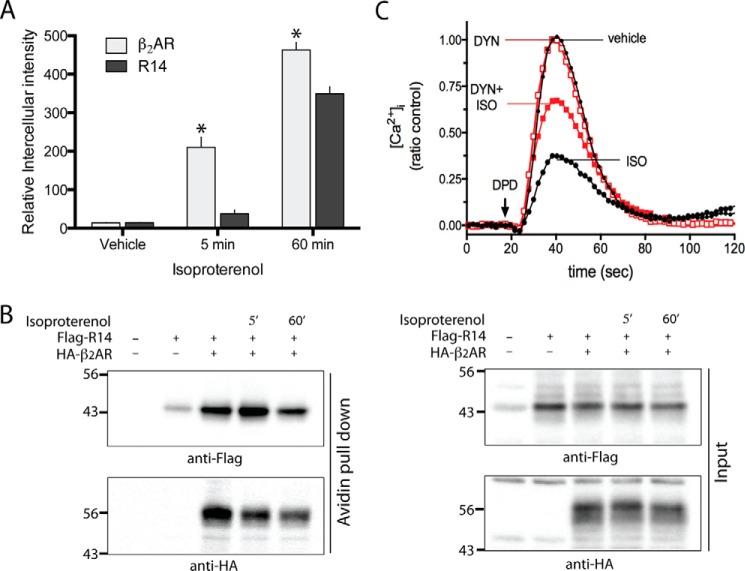
**Distribution and functional contributions of TAS2R14 internalization by β-agonist.** HEK-293T cells were transfected with HA-β_2_AR and FLAG-TAS2R14. Cells were treated with ISO for 5 min or 1 h, and the change in intracellular expression of each receptor determined by fluorescence microscopy (*A*) and the change in cell surface expression by the biotinylation assay (see “Experimental Procedures”) (*B*). As expected, intracellular β_2_AR increased in a time-dependent manner (*A*), with concomitant decrease in cell surface expression (*B*) after 5 min exposure to ISO. In contrast, there was no statistically significant redistribution of TAS2R14 from the cell surface to cytosol at 5 min. However, by 1 h intracellular accumulation and loss of cell surface TAS2R14 was detected. Results are representative of 5 independent experiments, where β_2_AR cell surface expression loss was 52 ± 2.1 and 86.75 ± 1.1%, and TAS2R14 loss was 11 ± 1.3 and 64 ± 2.6%, at the 5-min and 1-h time points, respectively. In *C*, the response to TAS2R14 agonist DPD is quantitated after 1 h of ISO in the absence or presence of the internalization inhibitor dynasore. The loss-of-function of TAS2R14 from 1 h ISO was only partially rescued by blocking internalization, consistent with the rapid receptor:receptor uncoupling process still in effect. Results are from a single representative experiment performed in triplicate. See text for statistical analysis from multiple experiments.

Interestingly, β_2_AR internalization from the cell surface to the interior is also underway after 5 min of agonist exposure (11, 12). We considered, then, that within the context of the heterodimer, TAS2R14 may co-internalize with β_2_AR, thereby resulting in an overall decreased cellular response to TAS2R14 agonist. However, whereas loss of β_2_AR cell surface expression was clearly apparent after 5 min of ISO exposure, TAS2AR14 cell surface expression was not changed (Fig. 6). In quantitative imaging studies, there was a readily detectable increase in intracellular β_2_AR after 5 min of treatment with agonist, and no statistically significant parallel increase in intracellular TAS2R14 at this time point (Fig. 7*A*). After a 1-h exposure to ISO, both β_2_AR and TAS2R14 intracellular expressions were increased, with β_2_AR > TAS2R14 (Figs. 6 and 7*A*). These imaging studies were confirmed using the cell surface biotinylation assay. In these assays we found β_2_AR cell surface loss was rapid and amounted to >25%, whereas TAS2R14 cell surface loss was minimally detected at 5 min. However, by 1-h ISO exposure, TAS2R14 cell surface expression was clearly decreased and approached >50% loss (Fig. 7*B*). So, at least with brief β-agonist exposure, the evidence does not support co-internalization. However, by 1-h ISO exposure, there was detectable loss of cell surface expression of TAS2R14, measured as a gain of intracellular expression (Figs. 6 and 7*A*), or loss of cell surface immunoreactivity in the biotinylation assays (Fig. 7*B*). This suggested that internalization of TAS2R14 during prolonged β-agonist exposure could be the basis for the further loss of cellular responsiveness to TAS2R14 agonist at this more prolonged time point. To further explore this, we blocked receptor internalization with the dynamin inhibitor dynasore. As shown in Fig. 7*C*, dynamin inhibition only partially rescued TAS2R14 desensitization by β-agonist after a 1-h exposure, indicating two processes at play: an early event that may be uncoupling of TAS2R14 to its G-protein due to interactions within the heterodimer, and a later internalization of TAS2R14 such that cell surface expression is reduced.

**FIGURE 8.**
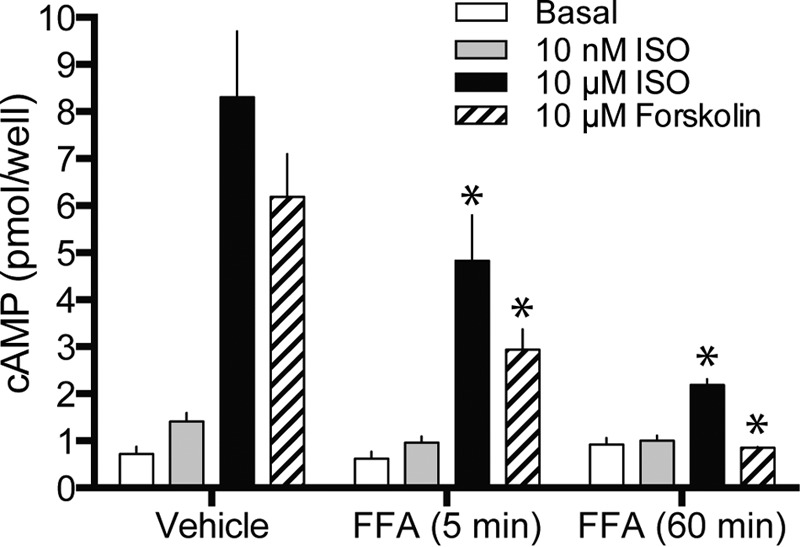
**Desensitization of adenylyl cyclase activity by TAS2R14 activation.** HASM cells were treated with 500 μm FFA for 5 min or 1 h, and then exposed to the indicated cAMP-stimulation agents in the presence of phosphodiesterase inhibition. β_2_AR (ISO)- and adenylyl cyclase-stimulated (forskolin) cAMP levels were decreased at both time points. When corrected for the decrease in forskolin-stimulated cAMP, β_2_AR-specific function was found to be unaffected by TAS2R14 agonism (see text). *, *p* < 0.01 *versus* vehicle, *n* = 11 (5 min) or 3 (1 h).

We next addressed the reverse scenario, where TAS2R14 conformation is altered by pretreatment with its agonist, and then β_2_AR function ascertained by measuring the cAMP response to ISO. With 5 min pre-treatment with FFA, ISO-stimulated cAMP was depressed (Fig. 8). However, this decrease was also observed with forskolin-stimulated cAMP (which increases cAMP by direct activation of adenylyl cyclase). With vehicle treatment, the ratio of the ISO response to the forskolin response was 1.3 ± 0.20, and with 5 min of treatment with DPD, the ratio was 1.5 ± 0.15 (*p* > 0.05). With the 1-h treatment with DPD, both ISO- and forskolin-stimulated cAMP levels were further depressed, and when corrected for the decreased forskolin response the ISO response was not impaired compared with vehicle treatment (Fig. 8). We thus conclude that there is no direct receptor:receptor cross-talk between activated TAS2R and β_2_AR, but rather a heterologous regulation distal to the receptor.

##### Physiological Consequences of the β_2_AR-TAS2R14 Interaction

**FIGURE 9.**
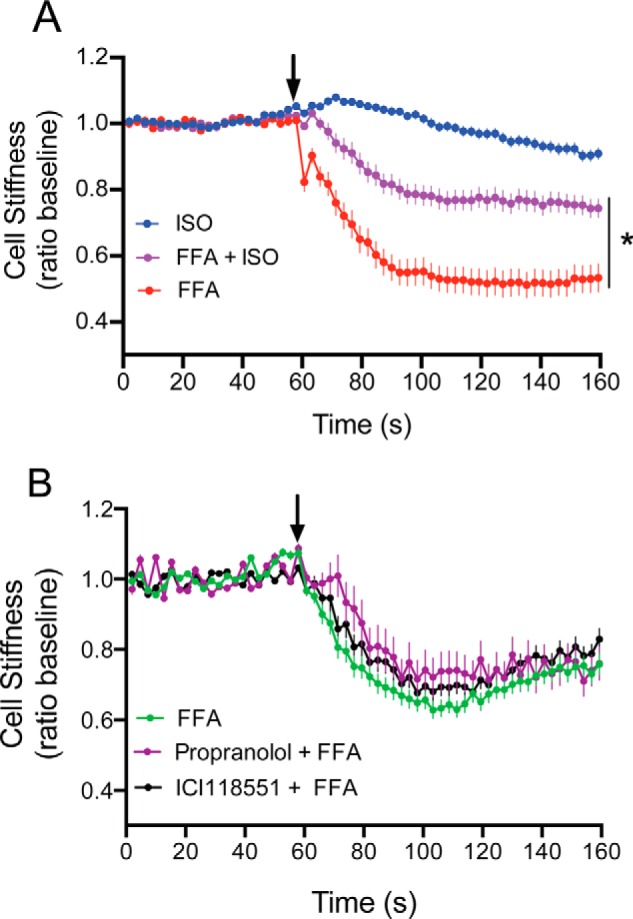
**Physiologic consequences of TAS2R:β_2_AR cross-talk in airway smooth muscle.** HASM cells were studied with magnetic twisting cytometry, which measures cell stiffness, where a decrease in stiffness in response to an agonist is the correlate to airway relaxation. In *A*, cells were treated with 10 μm ISO, 100 μm FFA, or ISO + FFA. All treatment conditions resulted in a decrease in cell stiffness compared with baseline (*p* < 0.001). However, maximal relaxation was not additive when both agonists were used. The response to the combination was less than when cells were treated with FFA alone. *, response less than FFA alone, *p* < 0.001. Data shown are results from 104 to 390 individual cell measurements from 3 independent experiments. In *B*, cells were exposed to FFA in the absence or presence of the neutral βAR antagonist propranolol (10 μm) or the β_2_AR inverse agonist ICI118551 (10 μm). The FFA-promoted relaxation response was unaffected by either agent. Results are from 231–272 individual cell measurements from 3 independent experiments.

Given the loss of TAS2R function evoked by activation of β_2_AR as measured by [Ca^2+^]*_i_*, we tested the relevance to ASM relaxation by studying single-cell mechanics with cultured HASM cells using magnetic twisting cytometry (3, 13, 14). Here, ferrimagnetic beads are attached to cell surface integrin receptors and perturbed magnetic fields serve to quantify increased or decreased cell stiffness (analogous to contraction and relaxation, respectively). Both β_2_ARs and TAS2Rs relax HASM. Because they do so by independent mechanisms, if there was no interaction we would expect an additive degree of relaxation when both receptors are activated, or, at least an equivalent degree (if at the maximal possible response). However, if β_2_ARs interact with TAS2Rs to decrease TAS2R function, then β-agonist would depress TAS2R14 agonist-mediated relaxation. The results of these physiologic studies are shown in Fig. 9*A*, and indeed show an impairment of TAS2R14 function when β_2_AR is activated by ISO. Relaxation to FFA was impaired by ∼50%, which is remarkably similar in degree to the loss of TAS2R14-stimulated [Ca^2+^]*_i_* observed in the cell-based studies (Fig. 5*A*). We also utilized this physiologic readout to ascertain if there was concordance with the [Ca^2+^]*_i_* results (Fig. 5*C*) observed when the β_2_AR is engaged by the neutral antagonist propranolol or the inverse agonist ICI118551. As shown in Fig. 9*B*, neither agent altered HASM relaxation by the TAS2R14 agonist FFA, consistent with the [Ca^2+^]*_i_* studies. Taken together, these results further strengthen the notion that the agonist-bound conformation of the β_2_AR is required to promote TAS2R14 dysfunction.

##### Specificity of the β_2_AR-TAS2R14 Interaction

**FIGURE 10.**
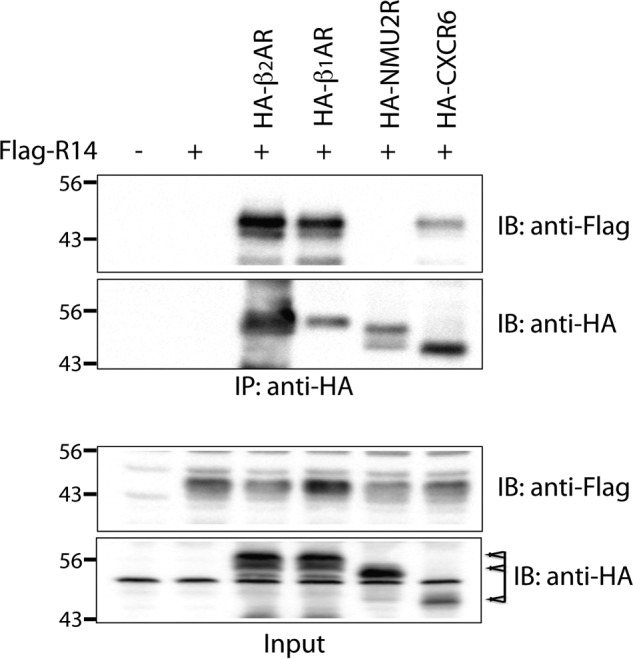
**TAS2R14 co-immunoprecipitation studies with other transfected GPCRs.** FLAG-TAS2R14 was transfected into HEK293T cells alone or with one of the following HA-tagged GPCRs: β_1_AR, NMU2R, or CXCR6. Lysates were immunoprecipitated with HA and immunoblotted with FLAG. The *upper section* shows co-immunoprecipitation of β_2_AR and β_1_AR with TAS2R14. No (or very little) co-immunoprecipitation was observed with HA-NUMU2R or HA-CXCR6 transfection. Results are representative of 4 independent experiments.

**FIGURE 11.**
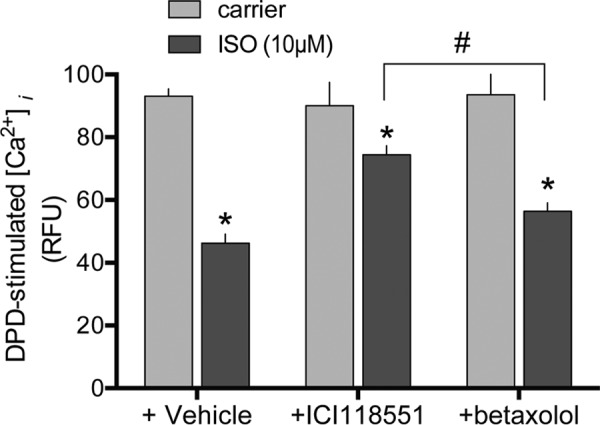
**Effects of β_1_AR or β_2_AR activation on TAS2R14 signaling to [Ca^2+^]*_i_*.** H1299 cells were pretreated with carrier, the β_2_AR antagonist ICI118551 (10 μm), or the β_1_AR antagonist betaxolol (10 μm) for 10 min, and then cells were treated for 5 min with 10 μm ISO. (The latter two conditions selectively activate β_1_AR and β_2_AR, respectively.) Desensitization of TAS2R14 signaling was observed with activation of either βAR subtype, and was greater when β_2_AR was activated compared with β_1_AR. *, *p* < 0.01 *versus* carrier; #, *p* < 0.01 + ICI118551 *versus* + betaxolol. Results are from 4 experiments.

We next tested whether the closely related β_1_AR subtype formed a complex with TAS2R14, and, whether β_1_AR activation altered TAS2R14 function. For the co-immunoprecipitation experiments, two phylogenetically distant GPCRs (15), the neuropeptide receptor NMU2R and the chemokine receptor CXCR6, were also studied. HEK-293T cells were transfected with FLAG-tagged TAS2R14 in the absence or presence of HA-tagged β_2_AR, β_1_AR, NMU2R, or CXCR6, immunoprecipitated with HA antibody, and immunoblotted with anti-FLAG antibodies (Fig. 10). As shown, β_1_AR, as well as the previously shown β_2_AR, formed complexes with TAS2R14 (*top panel*). In contrast, there was no signal for NMU2R and a minimally detected signal for CXCR6. The other panels are controls for the immunoprecipitation (*second panel*) and the inputs to the immunoprecipitation reaction (*bottom two panels*). These results suggested, then, that β_1_AR activation might also desensitize TAS2R14, as was seen with β_2_AR. Because human ASM do not express β_1_AR, we utilized the H1299 cell line that expresses β_1_AR, β_2_AR, and TAS2R14 (as determined by quantitative PCR, data not shown). Cells were treated with carrier (control) and ISO with pretreatment with vehicle, the β_1_AR antagonist betaxolol, or the β_2_AR antagonist ICI118551. The latter two conditions isolate β_2_AR and β_1_AR activation, respectively. After 5 min of agonist exposure, cells were treated with DPD and [Ca^2+^]*_i_* and immediately recorded (Fig. 11). There was a small (17%) but statistically significant desensitization of TAS2R14 function under the conditions of β_1_AR activation. When β_2_AR was selectively activated, there was a greater decrease in TAS2R14 function, amounting to ∼50% (see Fig. 11).

## Discussion

Here we show an interaction between TAS2R14 and β_2_AR on two levels. First, β_2_AR form heterodimers with TAS2R14 in the cytosol, which promotes expression of TAS2R14 on the cell membrane. This phenomenon was observed in heterologously expressing cells where receptors were transfected singly or together, and the expression phenotype was also found in cells that endogenously express both receptors where β_2_AR expression was depressed by shRNA, and in ASM cells from gene-ablated mice lacking β_1_- and β_2_AR expression. The data were highly consistent using these multiple approaches and cell types. The observation that even intracellular TAS2R14 expression is increased when β_2_AR are co-expressed suggests that the heterodimer stabilizes an otherwise unstable and readily degraded TAS2R14. Our results have some similarities to what has been reported with the mouse odorant receptor (OR) M71 (16). This receptor could not be readily expressed by heterologous transfection, remaining trapped in the cytosol. Co-transfection of β_2_AR was found to promote OR M71 expression via the formation of heterodimers. Although TAS2Rs and ORs are both chemoreceptors for exogenous substances involved in bitter taste and smell perception, respectively, they are not in the same family within the GPCR superfamily as indicted by the GRAFS classification system (15). ORs are part of the large rhodopsin family, whereas TAS2Rs are in the Frizzled family, one of four non-rhodopsin families. This suggests that this chaperone phenotype of β_2_AR is unlikely to be due to a highly specific amino acid sequence, given the difference in primary sequence between the two families. Nevertheless, a general region of the β_2_AR is likely involved. This notion is consistent with our finding that TAS2R14 also forms complexes with the related β_1_AR (which has a 72% amino acid identity in the transmembrane domains with β_2_AR), but not NMU2R (26% identify) or CXCR6 (19% identity). Of note, TAS2Rs have small extracellular N termini and intracellular C termini, as well as the loops that interconnect the transmembrane domains. Thus these compact receptors may require heterodimer formation with the dissimilar β_2_AR (longer N and C termini and third intracellular loop) to gain cell surface expression. Unlike the marked divergence in protein sequences and primary coupling pathways between TAS2R14 and β_2_AR, heterodimer formation between very closely related GPCR subtypes has also been reported (17, 18), including the facilitation of cell surface expression of a cytosol-locked receptor (17). For example, the α_1B_AR forms a heterodimer with the α_1D_AR, which results in cell surface expression of the latter (17). In this instance, specific amino acid residues may be responsible for the interaction of such closely related receptors.

Interestingly, for transfected TAS2R14, there is some level of expression in HEK-293T cells in the absence of co-transfecting β_2_AR. However, this cell line does express endogenous β_2_AR, which may act to provide some degree of expression of the transfected TAS2R14. Similarly, we do not obtain complete elimination of β_2_AR in the shRNA studies, which may explain detectable TAS2R14 signals in this setting. However, in the βAR-KO mouse ASM cells, there is no detectable β_1_- or β_2_AR, yet we repeatedly detect a low TAS2R14 signal. This may suggest that a small portion of intracellular TAS2R14 is ultimately inserted into the membrane in the absence of the β_2_AR chaperone effect, or, that another GPCR can also perform this function. In the context of the potential use of TAS2R agonists as bronchodilators, these expression data collectively indicate that measures to maintain β_2_AR expression would be beneficial in attaining higher TAS2R14 expression so as to obtain maximal airway relaxation.

However, β_2_AR acts as a double-edged sword once TAS2R14 is expressed in the cell membrane. The TAS2R14:β_2_AR heterodimer remains intact at rest, and activation of the β_2_AR component leads to rapid uncoupling of TAS2R14 to its signal transduction pathway. This effect is not due to generation of the β_2_AR second messenger cAMP, or co-internalization of TAS2R14, but appears to be due to induction of an unfavorable conformation of the TAS2R14 transmitted from the agonist-bound conformation of the β_2_AR. With prolonged β-agonist exposure, the uncoupling process remains in play, and internalization of TAS2R14 is observed, which further contributes to a loss of cellular responsiveness. Although most β_2_AR stabilize a similar set of conformations, there is evidence from cell-based and biophysical studies, of atypical conformations, which are nevertheless, coupled to G_s_/cAMP when bound to certain agonists (19, 20). Thus from a therapeutic standpoint, specific β_2_AR agonist/TAS2R14 agonist combinations would need to be tested to ascertain potential interactions of the two receptors.

The combined effects of expression modulation and coupling efficiency that β_2_AR has on TAS2R14 indicate a mechanism by which TAS2R14 functional responses can be regulated in a given cell. Although it has been suggested that proteins such as Ric8b (a putative GEF) may increase OR and TAS2R expression, the effect appears to be small (9). We propose that β_2_AR expression represents a major mechanism of stabilizing TAS2R14 in the cell and insertion into the membrane, via formation of a heterodimer. This mechanism is not restricted to overexpressing cells, but is clearly shown to be at play in endogenously expressing cells, and, to have physiological relevance. The consequences of *activated* (*i.e.* agonist occupied) β_2_AR on quenching TAS2R14 function has a mitigating effect on β_2_AR-mediated enhanced expression of TAS2R14. The ultimate impact of this cross-talk is dependent on the number of “spare receptors,” and, the read-out that is measured. In our studies with ASM cells, which express relatively low levels of TAS2R14 that cause a significant physiologic response, we have considered that there are few spare receptors and a high degree of amplification from receptor to ASM relaxation (3, 4). Thus the impact of the β-agonist-induced uncoupling of TAS2R14 is readily observed, amounting to ∼50% loss of function.

In summary, we have delineated a mechanism whereby β_2_AR promotes cell surface expression of the predominately intracellular TAS2R14. This function is due to formation of a β_2_AR:TAS2R14 heterodimer. Once inserted in the membrane, the TAS2R14 is functional, coupling to the canonical pathway, an increase in [Ca^2+^]*_i_*. In the steady-state, we propose that this form of the receptor (the heterodimer) achieves maximal function. However, when the conformation of the β_2_AR within the heterodimer is altered by β-agonist, there is a communication to TAS2R14 resulting in altered conformation, which is less favorable for coupling, leading to a loss of TAS2R14 function. This complex two-pronged interaction has implications in physiologic conditions/diseases where agonists for both β_2_AR and TAS2R14 might be utilized therapeutically.

## Experimental Procedures

### 

#### 

##### Cell Culture

HEK-293T were maintained in Dulbecco's modified Eagle's medium (DMEM) containing 10% fetal bovine serum (FBS), 100 units/ml of penicillin, and 100 μg/ml of streptomycin. Primary ASM cells were derived as previously described (21, 22) or obtained from Lonza (lot number 7F3984), maintained in SmBM media plus the SmGM-2 SingleQuot with growth factors (Lonza), and utilized at passages 3–8. H292 and H1299 cells were grown in RPMI 1640 in 10% FBS. HEK-293T, H292, and H1299 cell lines were obtained from ATCC. Cells were maintained at 37 °C, in a 95% air, 5% CO_2_ incubator, including periods when drugs were added to the cells. For the 96-well plate-based [Ca^2+^]*_i_* studies, cells were seeded at 40,000–80,000 cells/well the night before assays, and studied the next day at confluence.

##### cDNA Constructs and Transfections

The TAS2R14 cDNAs were subcloned into a pcDNA3.1 (HindIII-XboI) construct by PCR amplification, which included (5′ to 3′) the in-frame coding sequences for the Lucy peptide (MRPQILLLLALLTLGLA) (9), as well as the following: the FLAG tag, the extracellular N-terminal portion (first 45 amino acids of the coding sequence) of the rat somatostatin-3 receptor, TAS2R14, and a 12-amino acid epitope of HSV as described (2). pBiFC-VN155 and pBiFC-VC155 were obtained from Addgene. The β_2_AR-VN and TAS2R14-VC constructs were subcloned (EcoRI-XhoI) into pBiFC-VN155 and pBiFC-VC155, respectively. For co-IP studies, HEK-293T cells were transiently transfected using Lipofectamine 2000 (Invitrogen) and used for studies 36–48 h later. In other studies, cells were also transfected with Gα16/G44 to facilitate TAS2R14 signaling to [Ca^2+^]*_i_*, or G_gust_ (GNAT3), the canonical G-protein for TAS2Rs. For confocal imaging studies, HEK-293T cells were transiently transfected and then passaged to coverslips or chamber slides. For the BiFC studies, HEK-293T cells were transiently co-transfected with the indicated plasmids onto the Lab-TekII chamber slides (Fisher). For shRNA transfections, the following β_2_AR shRNA sequences were individually cloned into pLKO.1-pure: 5′-CCGGCCTCCTAAATTGGATAGGCTACTCGAGTAGCCTATCCAATTTAGGAGGTTTTT-3′; 5′-CCGGCCTCAAGACGTTAGGCATCATCTCGAGATGATGCCTAACGTCTTGAGGTTTTT-3′; 5′-CCGGGCCATCAACTGCTATGCCAATCTCGAGATTGGCATAGCAGTTGATGGCTTTTT-3′ (Sigma TRCN0000008084, TRCN0000008085, and TRCN0000008086, respectively). The control shRNA sequence was 5′-CCGGCAACAAGATGAAGAGCACCAACTCGAGTTGGTGCTCTTCATCTTGTTGTTTTT-3^1^ (Sigma SHC002). H292 were transfected with the three β_2_AR constructs or the control constructs using Lipofectamine 2000 with puromycin (7 μg/ml) selection to generate stable cell lines. TAS2R14, β_2_AR, and β-actin mRNA were determined by quantitative PCR using primers, conditions, and analysis exactly as previously described (3).

##### Intracellular Calcium Measurements

Measurements of [Ca^2+^]*_i_* mobilization in the aforementioned cell lines were determined as previously described (23, 24). Briefly, cells seeded in 96-well plates were loaded with the Ca^2+^-sensitive fluorescence indicator Fluo-4 (Life Technologies) and probenecid in Hanks' balanced salt solution, containing 1.3 mm CaCl_2_, 0.5 mm MgCl_2_·6H_2_O, 0.4 mm MgSO_4_·6H_2_O, 5.3 mm KCl, 0.4 mm KH_2_PO_4_, 4.2 mm NaHCO_3_, 137.9 mm NaCl, 0.3 mm Na_2_HPO_4_, 5.5 mm d-glucose, and 20 mm HEPES. Loading consisted of a 30-min incubation in a 5% CO_2_ atmosphere at 37 °C followed by a 30-min incubation at 25 °C at ambient atmosphere. The plate was read in a FlexStation3 plate reader (Molecular Devices), with excitation of 485 nm, emission of 525 nm, and a cut-off value of 515 nm. [Ca^2+^]*_i_*-stimulating agents or vehicle controls were robotically added at the 19-s point, and the fluorescent signal was measured every 1.52 s up to 120 s. In experiments with transiently transfected cells, [Ca^2+^]*_i_* measurements were made 36–48 h after transfection.

##### cAMP Measurements

cAMP was measured using a fluorescent competitive immunoassay (Molecular Devices) as previously described (12). Cells were cultured in 48-well plates and washed once with pre-warmed Krebs-Ringer bicarbonate buffer (10 mm glucose, 15 mm NaHCO_3_, pH 7.4). The cells were treated at 37 °C with 100 μm of the phosphodiesterase inhibitor 3-isobutyl-1-methylxanthine for 30 min, and then exposed to vehicle, or TAS2R14 agonist, for the indicated times. To stimulate cAMP, cells were then exposed to ISO (10 nm or 10 μm) or forskolin (10 μm) in the presence of 100 μm l-ascorbic acid for 30 min. Cells were lysed by the addition of a hypotonic buffer, and cAMP was measured on the FlexStation3, with concentrations determined using a standard curve.

##### Receptor and [Ca^2+^]_i_ Imaging

To localize epitope-tagged β_2_AR, TAS2R14, and cellular calcium, confocal microscopy was employed as previously described (12) with an Olympus FV1000 MPE multiphoton laser-scanning microscope. After transfection, HEK-293T cells were transferred onto 12-mm coverslips coated with poly-l-lysine in 6-well plates and studied 48 h later. In a typical localization experiment, attached HEK-293T cells maintained in DMEM were treated with agonist or vehicle for the indicated times at 37 °C in 5% CO_2_, washed three times with cold phosphate-buffered saline, and then fixed with 4% paraformaldehyde. β_2_AR receptors were identified with a mouse anti-HA (12CA4) primary antibody (Roche Applied Science) and TAS2R14 receptors with a rabbit anti-FLAG-M2 primary antibody (Sigma). Secondary antibodies were goat anti-rabbit antibody conjugated with Alexa Fluor 594 (Molecular Probes) and goat anti-mouse antibody conjugated with FITC (Sigma). Nuclei were identified with DAPI (Molecular Probes). For live cell calcium imaging, the transfected cells were transferred onto 8-well chamber slides, and 48 h later loaded with Fluo-4 loading followed by treatment with the indicated agonist or vehicle. Images were acquired using the EVOS Cell Imaging System (Thermo Fisher) with excitation at 488 nm and a 515–540 nm emission filter.

##### Co-immunoprecipitation and Western Blots

These studies were carried out using methods as previously described (18, 22) with modifications. Transfected cells were washed in ice-cold PBS and lysed in 1 ml of TNEN (100 mm Tris-Cl, 75 mm NaCl, 0.1 m EDTA, 0.5% Nonidet P-40, 0.3% Triton X-100 with proteinase inhibitor mixture) buffer. Cell debris was removed by centrifugation at 14,000 rpm for 25 min at 4 °C. Total cell lysate (400 μg) was pre-cleared by incubation with protein A/G (2:1)-agarose beads (Gibco BRL) for 30 min, centrifuged to remove the beads, and incubated with primary antibody overnight at 4 °C. Protein A/G beads were then added, and the suspension was incubated at 4 °C for 2 h with constant rotation. The beads were washed four times with TNEN, and proteins were released by addition of 2× Laemmli sample buffer. Equivalent amounts of eluted proteins were subjected to SDS-PAGE, transferred to PVDF (Millipore) membranes, and immunoblots were performed with the indicated antibodies. Bands were visualized using chemiluminescence (Thermo Scientific, 1:1000 secondary antibody titer) and detection by the ChemiDoc MP imaging system (Bio-Rad). Bands were quantitated using the provided software or Image-J (National Institutes of Health). For certain experiments, a membrane protein extraction system (Mem-PER, Thermo Scientific) was utilized to separate membrane and cytosolic cell fractions. Briefly, transfected HEK-293T were detached and washed by centrifugation, and 0.75 ml of permeabilization buffer added to the pellet. Cells were incubated for 20 min at 4 °C with constant rotation. Permeabilized cells were centrifuged for 15 min at 16,000 rpm, and the supernatant (cytosolic fraction) was removed. The pellet (membrane fraction) was solubilized with 0.5 ml of the included solubilization buffer for 30 min at 4 °C, and was clarified by centrifugation. For standard immunoblotting 8–10 μg of each fraction was used, whereas 200–250 μg was used for immunoprecipitation. For these and other studies, protein concentrations were determined by the method of Bradford (25). For the biotinylation studies, a cell surface protein isolation system (Pierce, Thermo Scientific) was utilized. Briefly, cells in a 10-cm culture dish were washed with PBS and then 10 ml of biotin solution consisting of 12 mg of sulfo-NHS-SS-Biotin in 50 ml of cold PBS was added to the dish, which was then rocked on an orbital shaker for 1 h at 4 °C. The reaction was stopped by addition of 500 μl of quenching solution. Cells were detached by scraping, washed twice by centrifugation, and resuspension in Tris-buffered saline, and then solubilized in 1 ml of RIPA buffer. Cell debris was removed by centrifugation at 14,000 rpm. To the supernatant, 60 μl of NeutrAvidin-agarose beads were added and incubated overnight at 4 °C. The beads were washed four times with lysis buffer and the proteins were released by addition of 2× Laemmli sample buffer. The catalogue numbers and sources for the antibodies were: anti-FLAG antibody (F7425, Sigma), anti-HA antibody (11-583-816001, Roche), anti-β-actin (A1978, Sigma), anti-Myc (SC4, Santa Cruz), anti-Na/K-ATPase (3010, Cell Signaling), anti-GAPDH (2118, Cell Signaling), anti-mouse conjugate FITC (AP127F, Sigma), anti-rabbit conjugate Alexa 594 (A11012, Thermo Fisher), anti-HA-agarose bead (A2095, Sigma, for IP), and anti-FLAG M2 magnetic beads (M8823, Sigma, for IP).

##### Magnetic Twisting Cytometry

The physiological consequences of the TAS2R14-β_2_AR interaction were ascertained by examining single cell mechanics of HASM using magnetic twisting cytometry (3, 13, 14, 24). These experiments were performed exactly as previously described (3, 13, 26). Briefly, RGD-coated ferrimagnetic microbeads were attached to cell surface integrin receptors, magnetized horizontally, and then twisted in a vertically aligned magnetic field. Lateral bead displacement measures smooth muscle “contraction” and “relaxation,” in response to the application of bronchoreactive drugs to the media, which correlates with airway constriction and dilation in the *ex vivo* and *in vivo* settings (3, 14).

##### Statistical Analyses

The data from the biochemical studies were analyzed by two-sided, paired or unpaired *t*-tests (as appropriate). Multiple comparisons were performed by two-way analysis of variance with post hoc *t*-tests adjusted by Bonferroni's method. For the magnetic twisting cytometry studies, nested design analysis was used as previously described (27), which controls for random effects of repeated measurements of multiple cells from the same flask. For all studies, significance was imparted when *p* < 0.05. Analysis was performed using SAS V9.2 (SAS Institute Inc.) and Prism (GraphPad). Results are shown as mean ± S.E.

## Author Contributions

D. K., S. S. A., and S. B. L. conceived and designed the research. D. K., S. H. P., and H. M. Y. performed the experiments. D. K., S. H. P., S. S. A., and S. B. L. analyzed the data and wrote the manuscript.

## Footnotes

2 The abbreviations used are:

TAS2R: human bitter taste receptor
β_2_AR: β_2_-adrenergic receptor
α_1_AR: α_1_-adrenergic receptor
GPCR: G-protein coupled receptor
ASM: airway smooth muscle
HASM: human airway smooth muscle
DPD: diphenhydramine
FFA: flufenamic acid
ISO: isoproterenol
BiFC: biomolecular fluorescence complementation
OR: odorant receptor
IP: immunoprecipitation
VN: amino terminal of Venus
VC: carboxyl terminus of Venus.
